# Crystal structure and Hirshfeld surface analysis of 3-({4-[(4-cyano­phen­oxy)carbon­yl]phen­oxy}carbon­yl)phenyl 4-(benz­yloxy)-3-chloro­benzoate

**DOI:** 10.1107/S2056989022008441

**Published:** 2022-09-08

**Authors:** S. Selvanandan, H. Anil kumar, H. T. Srinivasa, B. S. Palakshamurthy

**Affiliations:** a Department of Physics, ACS College of Engineering, Bangalore, Karnataka-560074, India; b Department of Physics, Government First Grade College, Magadi, Karnataka-562120, India; c Raman Research Institute, C. V. Raman Avenue, Sadashivanagar, Bangalore, Karnataka, India; d Department of PG Studies and Research in Physics, Albert Einstein Block, UCS, Tumkur University, Tumkur, Karnataka-572103, India; University of Aberdeen, Scotland

**Keywords:** crystal structure, Hirshfeld surface, energy framework

## Abstract

The title compound, C_35_H_22_ClNO_7_, is a non-liquid crystal with a bent-shaped mol­ecule. The dihedral angles between adjacent aromatic rings in the mol­ecule (starting from the cyano­benzene ring) are 72.61 (2), 87.69 (4), 64.08 (2) and 88.23 (2)°, indicating that adjacent rings are close to perpendicular to each other. In the crystal, the mol­ecules are linked by weak C—H⋯N and C—H⋯π inter­actions, thereby forming a two-dimensional supra­molecular architecture in the *ac* plane. The most important contributions to the crystal packing arise from H⋯H (59.3%), S⋯H (27.4%) and O⋯H (7.5%) inter­actions, as determined by a Hirshfeld surface analysis.

## Abstract 2

The title compound is a non-liquid crystal with a bent-shaped mol­ecule in which adjacent aromatic rings are close to perpendicular to each other.

## 1. Chemical context

Banana/bent-shaped liquid crystals (LCs) are of great inter­est in the field of display materials. In particular, the –CN groups at the terminal end (Walba *et al.*, 2000 ▸; Reddy & Sadashiva, 2004 ▸) of banana-shaped LCs have been linked to their bent or bow (twisted) anisometric phase with *C*_2*v*_ symmetry. Furthermore, they exhibit polar order, chirality and spontaneous polarization in the fluid phase. We have reported the crystal structures of LC inter­mediates and found that benz­yloxy group-substituted mol­ecules are prone to be hydro­phobic (Kashi *et al.*, 2012 ▸; Al-Eryani *et al.*, 2011 ▸). Benz­yloxy group-substituted mol­ecules also play a significant role in synthesizing bent-shaped LCs and non-LCs (Palakshamurthy *et al.*, 2012 ▸). Hence, it is useful to study benz­yloxy group-substituted bent-shaped mol­ecules to understand the structural properties and the relationship between LCs and crystal structures. 
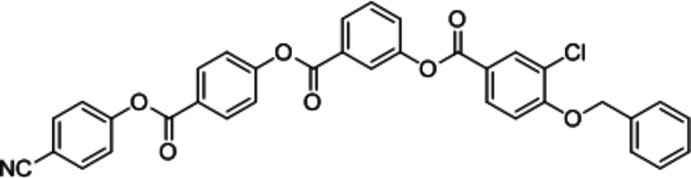


In a continuation of this work, we investigated the title mol­ecule, which possesses five aromatic rings with three ester groups and a benz­yloxy group at one terminal end, presumably making the mol­ecule highly polar. Furthermore, it has a chloro group at one side and a cyano group at the opposite terminal end of the mol­ecule, inducing an unsymmetrical structure (Hartung *et al.*, 2000 ▸). The mol­ecule was subjected to LC characterization studies, but it did not show any LC properties, which may be due to the absence of a flexible alkyl chain. The title compound was synthesized according to the procedure described by Sadashiva *et al.* (2002 ▸) and its crystal structure is reported herein.

## 2. Structural commentary

**Figure 1:**
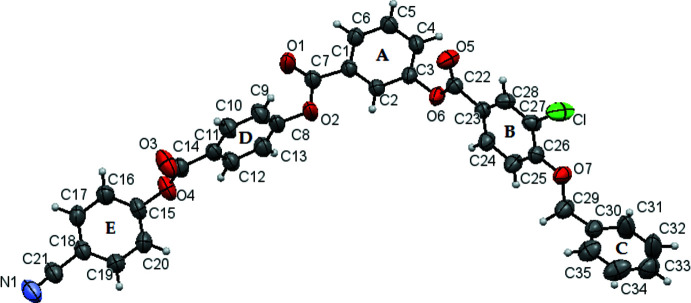
The mol­ecular structure of the title compound, showing displacement ellipsoids drawn at the 50% probability level.

The mol­ecular structure of the title compound is shown in Fig. 1 ▸. The dihedral angles between the aromatic rings are as follows: *A*/*B* = 64.08 (2), *A*/*C*= 29.75 (2), *A*/*D* = 87.69 (4), *A*/*E* = 16.07 (3), *B*/*C* = 88.23 (2), *B*/*D* = 87.88 (4), *B*/*E* = 68.87 (4), *C*/*D* = 82.27 (3), *E*/*D* = 72.61 (2) and *C*/*E* = 37.46 (4)°, where *A*, *B*, *C*, *D* and *E* are the C1–C6, C23–C28, C30–C35, C8–C13 and C15–C20, rings, respectively. The torsion angles associated with the benz­yloxy group are −7.2 (3) (C15—O4—C14—O3), −3.1 (3) (C8—O2—C7—O1) and −0.7 (2)° (C3—O6—C22—O5). Three short intra­molecular C—H⋯O contacts (Table 1 ▸) may influence the mol­ecular conformation.

## 3. Supra­molecular features

**Figure 2:**
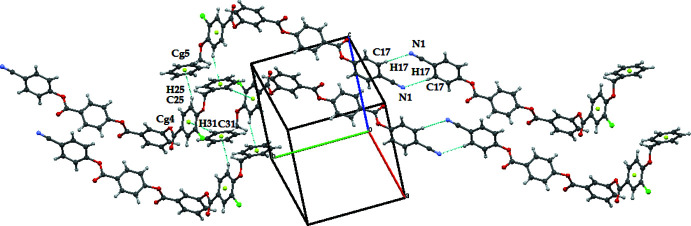
The mol­ecular packing of the title compound. Dashed lines indicate the C—H⋯π inter­actions.

**Figure 3:**
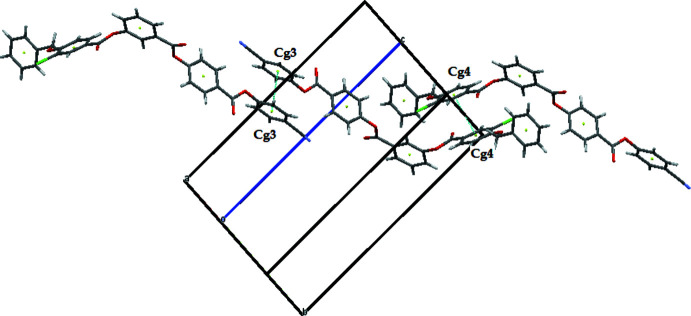
The mol­ecular packing of the title compound. Dashed lines indicate the π–π stacking inter­actions.

In the crystal, the mol­ecules are linked by weak C—H⋯N hydrogen bonds and weak C—H⋯π inter­actions (Table 1 ▸) to generate a two-dimensional supra­molecular architecture propagating in the *ac* plane as shown in Fig. 2 ▸. Furthermore, the mol­ecules are linked by centrosymmetric aromatic π–π stacking inter­actions with *Cg*4⋯*Cg*4 and *Cg*3⋯*Cg*3 = 3.6387 (10) Å (slippage = 1.086 Å) and 3.7740 (10) (slippage = 1.407 Å), respectively, as shown in Fig. 3 ▸ (*Cg*4 is the centroid of the C23–C28 ring and *Cg*3 is the centroid of the C15–C20 ring).

## 4. Database survey

A search of the Cambridge Structural Database (CSD, version 5.42, update of November 2020; Groom *et al.*, 2016 ▸) for mol­ecules containing the (4-cyano­phen­oxy)carbonyl fragment resulted in four matches with CSD refcodes EWUSIA (Srinivasa *et al.*, 2015 ▸), IBUXOV (Ji *et al.*, 2017 ▸), IBUXUB (Yingchun *et al.*, 2016 ▸) and OCUTIS (Yingchun *et al.*, 2016 ▸). In all these structures there is a 4-cyano­phen­oxy grouping at the one end of the mol­ecule, similar to the title compound. In IBUXOV, IBUXUB and OCUTIS the same core exists at both ends of the mol­ecule. Sometimes the presence of a –CN group at both terminals of the mol­ecule induces liquid-crystal properties.

In EWUSIA, the dihedral angles between the cyano­benzoate ring and the first neighbouring benzene ring, and between the second neighbour and the first and second benzene rings are 50.47 (2), 10.15 (3) and 50.02 (5)° compared to 72.61 (2), 16.06 (2) and 87.69 (4)° in the title mol­ecule. In IBUXOV, the dihedral angles between the rings (cyano­benzoate ring and the neighbouring benzene ring) are 69.45 (2) and 64.20 (3)°, and 73.60 (3) and 84.16 (3)° between the adjacent cyano­benzoate and benzene rings themselves. In IBUXUB, the dihedral angles between the rings (cyano­benzoate and the neighbouring benzene ring) are 69.68 (2) and 74.28 (4)°, and 48.87 (2) and 89.88 (4)° between the cyano­benzoate and benzene rings. In OCUTIS, the dihedral angles between adjacent cyano­benzoate and benzene rings are 81.21 (4) and 54.43 (2)° compared to angles between the cyano­benzoate and benzene rings of 55.02 (3) and 84.20 (3)°.

## 5. Hirshfeld surface analysis

**Figure 4:**
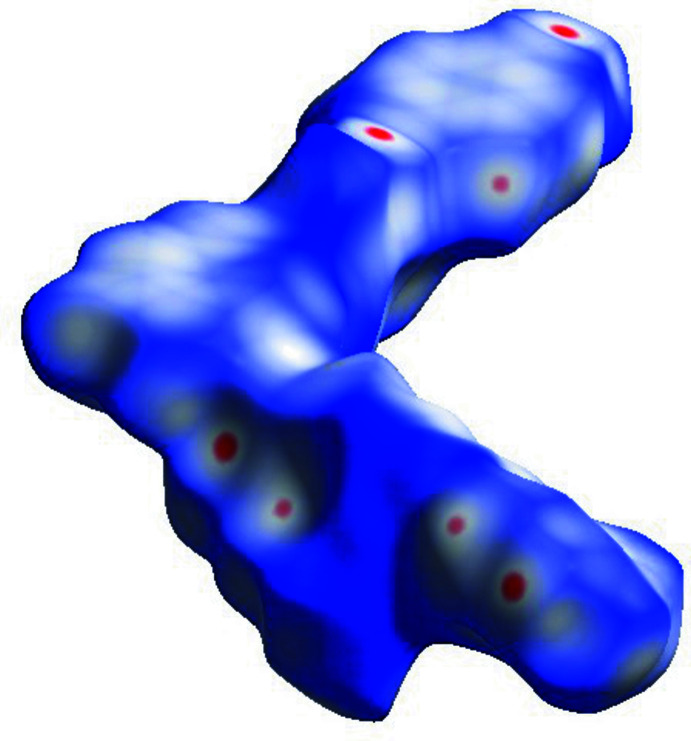
Hirshfeld surface of the title compound mapped with *d*_norm_.

**Figure 5:**
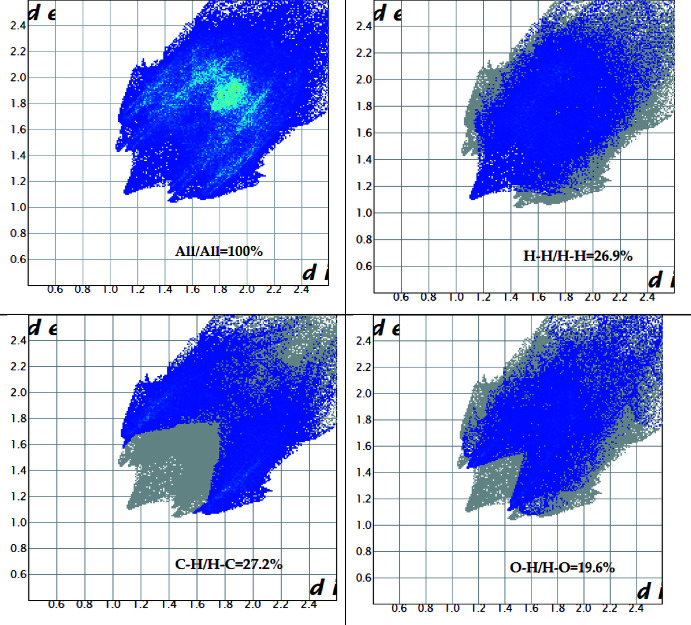
Two-dimensional fingerprint plots for the title compound.

**Figure 6:**
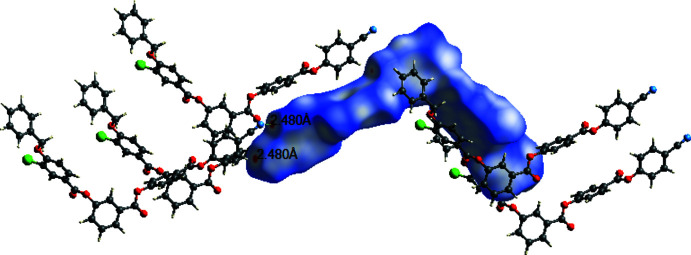
Hirshfeld surface of the title compound mapped over *d*_norm_, showing the C—H⋯N inter­actions.

*CrystalExplorer17.5* (Turner *et al.*, 2017 ▸) was used to perform the Hirshfeld surface analysis (Spackman & Jayatilaka, 2009 ▸) to further qu­antify the various inter­molecular inter­actions. The Hirshfeld surface mapped over *d*_norm_ is illustrated in Fig. 4 ▸ and the associated two-dimensional fingerprint plots in Fig. 5 ▸. The major contributions to the crystal structure are from H⋯H (26.9%), C⋯H (27.2%) and O⋯H (19.6%) contacts. In Figs. 6 ▸ and 7 ▸, the red spots on the *d*_norm_ and *d*_e_ surfaces represent the C—H⋯π inter­actions.

## 6. Synthesis and crystallization

4-[(4-Cyano­phen­oxy)carbon­yl]phenyl 3-hy­droxy­benzoate (1 mmol) and 4-(benz­yloxy)-3-chloro­benzoic acid (1.2 mmol) were dissolved in dry chloro­form (50 ml). After the addition of *N*,*N*-di­cyclo­hexyl­carbodi­imide (1.2 mmol) and a catalytic amount of 4-(*N*,*N*-di­methyl­amino)­pyridine (DMAP), the mixture was stirred at room temperature for about 12 h. The di­cyclo­hexyl­urea that precipitated was filtered off and the filtrate diluted with chloro­form. This solution was washed with 2% aqueous acetic acid solution (10 ml) and 5% ice-cold sodium hydroxide solution (10 ml) and finally washed with water and dried over anhydrous sodium sulfate. The crude residue obtained was chromatographed on silica gel using chloro­form as an eluent. Removal of solvent from the eluate afforded the white target material, which was crystallized from a mixture of chloro­form and aceto­nitrile. Single crystals in the form of colourless prisms suitable for diffraction studies were grown from a solution in ethyl alcohol by slow evaporation.

IR (nujol) λ_max_: 3105, 3080, 2237, 1738, 1733, 1614, 1523, 1452, 1253, 1054 cm^−1^; ^1^H NMR (500 MHz, CDCl_3_) *δ* H: 8.22 (*m*, 3H, Ar—H) , 8.19 (*m*, 3H, Ar-H), 8.02 (*m*, 2H, Ar—H), 7.98–7.30 (*m*, 7H, Ar—H), 6.99 (*m*, 5H, Ar—H), 5.22 (*s*, 2H, Ar—O—CH_2_–) ppm; ^13^C NMR (125 MHz, CDCl_3_) *δ*: 165.2, 159.8, 154.6, 153.7, 151.2, 136.7, 132.6, 130.2, 129, 128.9, 128.6, 127.6, 127.1, 126.8, 123.9, 122.3, 121.3, 112.4 ppm. Micro elemental analysis calculated for C_35_H_22_ClNO_7_; C, 69.60; H, 3.67; Cl, 5.87; N, 2.32; found C, 69.68; H, 3.72; Cl, 5.91; N, 2.35%.

## 7. Refinement

Crystal data, data collection and structure refinement details are summarized in Table 2 ▸. Atoms H2, H4 and H6 were fully refined. Other H atoms were positioned with idealized geometry and refined using a riding model with C—H = 0.93–0.97 Å and *U*_iso_(H) = 1.2–1.5*U*_eq_(C).

## Supplementary Material

Crystal structure: contains datablock(s) I. DOI: 10.1107/S2056989022008441/hb8019sup1.cif

Structure factors: contains datablock(s) I. DOI: 10.1107/S2056989022008441/hb8019Isup2.hkl

Supporting information file. DOI: 10.1107/S2056989022008441/hb8019Isup3.cml

CCDC reference: 2023150

Additional supporting information:  crystallographic information; 3D view; checkCIF report

## Figures and Tables

**Figure 7:**
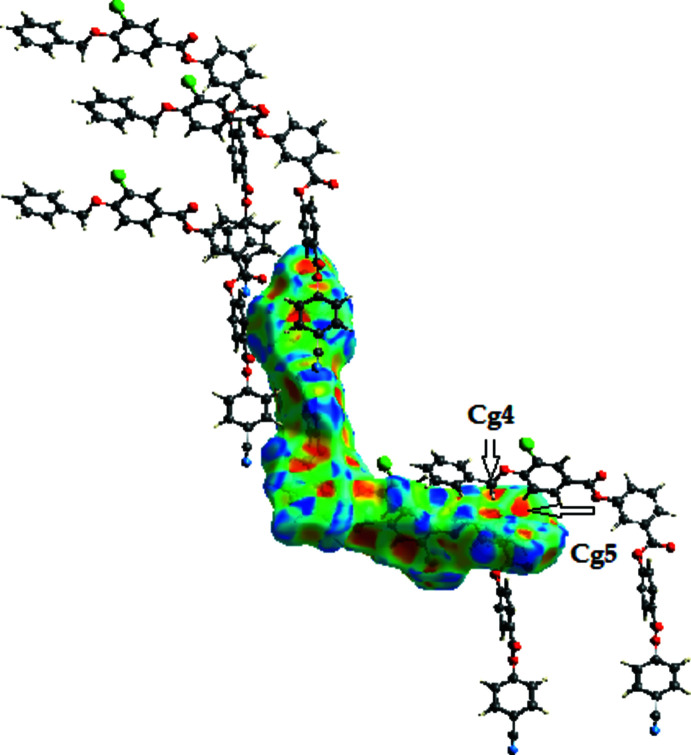
Hirshfeld surface of the title compound mapped over shape-index, showing the C—H⋯π inter­actions.

**Table 1:** Hydrogen-bond geometry (Å, °) *Cg*4 and *Cg*5 are the centroids of the C23–C28 and C30–C35 rings, respectively.

*D*—H⋯*A*	*D*—H	H⋯*A*	*D*⋯*A*	*D*—H⋯*A*
C2—H2⋯O2	0.944 (18)	2.411 (17)	2.7213 (19)	98.9 (12)
C12—H12⋯O4	0.93	2.42	2.733 (2)	100
C24—H24⋯O6	0.93	2.40	2.721 (2)	100
C17—H17⋯N1^i^	0.93	2.62	3.504 (3)	158
C25—H25⋯*Cg*5^ii^	0.93	2.86	3.744 (2)	158
C31—H31⋯*Cg*4^iii^	0.93	2.82	3.702 (3)	158

Symmetry codes: (i) 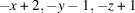 ; (ii) 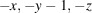 ; (iii) 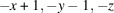 .

**Table 2:** Experimental details

Crystal data	
Chemical formula	C_35_H_22_ClNO_7_
*M*_r_	603.98
Crystal system, space group	Triclinic, *P* 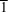
Temperature (K)	296
*a*, *b*, *c* (Å)	8.0202 (1), 9.8474 (2), 19.4712 (4)
α, β, γ (°)	95.422 (1), 94.693 (1), 103.857 (1)
*V* (Å^3^)	1477.66 (5)
*Z*	2
Radiation type	Mo *K*α
μ (mm^−1^)	0.18
Crystal size (mm)	0.19 × 0.18 × 0.16

Data collection	
Diffractometer	Bruker SMART APEXII CCD
Absorption correction	Multi-scan (*SADABS*; Bruker, 2017 ▸)
*T*_min_, *T*_max_	0.966, 0.971
No. of measured, independent and observed [*I* > 2σ(*I*)] reflections	25466, 5207, 4255
*R*_int_	0.024
(sin θ/λ)_max_ (Å^−1^)	0.595

Refinement	
*R*[*F* ^2^ > 2σ(*F* ^2^)], *wR*(*F* ^2^), *S*	0.038, 0.114, 1.03
No. of reflections	5207
No. of parameters	410
No. of restraints	6
H-atom treatment	H atoms treated by a mixture of independent and constrained refinement
Δρ_max_, Δρ_min_ (e Å^−3^)	0.26, −0.33

Computer programs: *APEX2* and *SAINT* (Bruker, 2017 ▸), *SHELXT* (Sheldrick, 2015*a* ▸), *SHELXL* (Sheldrick, 2015*b* ▸) and *Mercury* (Macrae *et al.*, 2020 ▸).

## supplementary crystallographic information

### Crystal data

**Table:**

C_35_H_22_ClNO_7_	*F*(000) = 624
*M**_r_* = 603.98	Prism
Triclinic, *P*1	*D*_x_ = 1.357 Mg m^−^^3^
Hall symbol: -P 1	Melting point: 445 K
*a* = 8.0202 (1) Å	Mo *K*α radiation, λ = 0.71073 Å
*b* = 9.8474 (2) Å	Cell parameters from 5212 reflections
*c* = 19.4712 (4) Å	θ = 1.0–25.0°
α = 95.422 (1)°	µ = 0.18 mm^−^^1^
β = 94.693 (1)°	*T* = 296 K
γ = 103.857 (1)°	Prism, colourless
*V* = 1477.66 (5) Å^3^	0.19 × 0.18 × 0.16 mm
*Z* = 2	

### Data collection

**Table:**

Bruker SMART APEXII CCD diffractometer	5207 independent reflections
Radiation source: fine-focus sealed tube	4255 reflections with *I* > 2σ(*I*)
Graphite monochromator	*R*_int_ = 0.024
Detector resolution: 2.06 pixels mm^-1^	θ_max_ = 25.0°, θ_min_ = 2.1°
ω scans	*h* = −9→9
Absorption correction: multi-scan (SADABS; Bruker, 2017)	*k* = −11→11
*T*_min_ = 0.966, *T*_max_ = 0.971	*l* = −23→23
25466 measured reflections	

### Refinement

**Table:**

Refinement on *F*^2^	Secondary atom site location: difference Fourier map
Least-squares matrix: full	Hydrogen site location: mixed
*R*[*F*^2^ > 2σ(*F*^2^)] = 0.038	H atoms treated by a mixture of independent and constrained refinement
*wR*(*F*^2^) = 0.114	*w* = 1/[σ^2^(*F*_o_^2^) + (0.0619*P*)^2^ + 0.2788*P*] where *P* = (*F*_o_^2^ + 2*F*_c_^2^)/3
*S* = 1.03	(Δ/σ)_max_ = 0.002
5207 reflections	Δρ_max_ = 0.26 e Å^−^^3^
410 parameters	Δρ_min_ = −0.32 e Å^−^^3^
6 restraints	Extinction correction: SHELXL2018 (Sheldrick, 2015b), Fc^*^=kFc[1+0.001xFc^2^λ^3^/sin(2θ)]^-1/4^
0 constraints	Extinction coefficient: 0.0158 (18)
Primary atom site location: structure-invariant direct methods	

### Special details

**Table:**

Geometry. All esds (except the esd in the dihedral angle between two l.s. planes) are estimated using the full covariance matrix. The cell esds are taken into account individually in the estimation of esds in distances, angles and torsion angles; correlations between esds in cell parameters are only used when they are defined by crystal symmetry. An approximate (isotropic) treatment of cell esds is used for estimating esds involving l.s. planes.

### Fractional atomic coordinates and isotropic or equivalent isotropic displacement parameters (Å^2^)

**Table:**

	*x*	*y*	*z*	*U*_iso_*/*U*_eq_	
O7	0.66155 (14)	1.37557 (12)	1.06583 (6)	0.0604 (3)	
O6	0.23387 (14)	0.91624 (11)	0.82866 (6)	0.0562 (3)	
O2	0.31260 (15)	0.47851 (13)	0.70608 (7)	0.0701 (4)	
O4	0.70509 (18)	0.04285 (14)	0.58733 (6)	0.0691 (4)	
O5	0.07952 (16)	0.85138 (14)	0.91636 (6)	0.0683 (4)	
O3	0.6173 (2)	−0.06006 (17)	0.67969 (8)	0.0920 (5)	
O1	0.06206 (16)	0.37006 (13)	0.64386 (7)	0.0734 (4)	
N1	1.2423 (3)	−0.3715 (2)	0.52599 (11)	0.0906 (6)	
C33	1.1312 (3)	1.7706 (3)	1.18897 (11)	0.0805 (6)	
H33	1.200300	1.842548	1.220743	0.097*	
C34	1.1522 (3)	1.6373 (3)	1.18768 (11)	0.0812 (6)	
H34	1.235250	1.618164	1.218912	0.097*	
C35	1.0499 (3)	1.5295 (2)	1.13982 (10)	0.0710 (5)	
H35	1.066616	1.439064	1.138300	0.085*	
C30	0.9239 (2)	1.55720 (18)	1.09471 (8)	0.0561 (4)	
C29	0.8184 (2)	1.44647 (19)	1.03988 (9)	0.0652 (5)	
H29A	0.790624	1.489441	0.999036	0.078*	
H29B	0.883975	1.378979	1.026758	0.078*	
C26	0.5546 (2)	1.27075 (17)	1.02142 (8)	0.0496 (4)	
C25	0.5822 (2)	1.23457 (18)	0.95365 (9)	0.0599 (4)	
H25	0.678848	1.285319	0.935657	0.072*	
C24	0.4681 (2)	1.12408 (17)	0.91246 (8)	0.0550 (4)	
H24	0.488830	1.101130	0.867042	0.066*	
C23	0.32400 (19)	1.04740 (15)	0.93774 (8)	0.0456 (3)	
C22	0.19929 (19)	0.92835 (16)	0.89583 (8)	0.0472 (4)	
C3	0.1246 (2)	0.80725 (15)	0.78186 (8)	0.0476 (4)	
C2	0.1912 (2)	0.69862 (16)	0.75737 (8)	0.0479 (4)	
C1	0.08826 (19)	0.59329 (16)	0.70854 (7)	0.0449 (3)	
C7	0.1464 (2)	0.46904 (17)	0.68174 (8)	0.0517 (4)	
C8	0.3800 (2)	0.36353 (18)	0.68774 (9)	0.0577 (4)	
C13	0.4822 (2)	0.37070 (19)	0.63451 (10)	0.0608 (4)	
H13	0.499046	0.447392	0.609152	0.073*	
C12	0.5600 (2)	0.26220 (18)	0.61907 (9)	0.0572 (4)	
H12	0.630774	0.266133	0.583388	0.069*	
C11	0.53291 (19)	0.14754 (17)	0.65663 (8)	0.0494 (4)	
C14	0.6184 (2)	0.03171 (19)	0.64397 (9)	0.0551 (4)	
C15	0.8124 (2)	−0.04890 (18)	0.57562 (9)	0.0566 (4)	
C20	0.9844 (2)	0.00167 (19)	0.59635 (10)	0.0654 (5)	
H20	1.027213	0.091840	0.619681	0.078*	
C19	1.0936 (2)	−0.08343 (19)	0.58204 (10)	0.0657 (5)	
H19	1.211388	−0.050869	0.595875	0.079*	
C18	1.0288 (2)	−0.21700 (18)	0.54721 (9)	0.0564 (4)	
C21	1.1458 (3)	−0.3048 (2)	0.53442 (10)	0.0671 (5)	
C27	0.40785 (19)	1.19401 (19)	1.04630 (8)	0.0526 (4)	
C28	0.29494 (19)	1.08363 (18)	1.00565 (8)	0.0530 (4)	
H28	0.198360	1.032683	1.023589	0.064*	
C9	0.3515 (2)	0.2511 (2)	0.72563 (10)	0.0674 (5)	
H9	0.281420	0.248249	0.761511	0.081*	
C10	0.4279 (2)	0.1426 (2)	0.70986 (9)	0.0615 (4)	
H10	0.408968	0.065641	0.735040	0.074*	
C17	0.8540 (2)	−0.26542 (19)	0.52624 (9)	0.0621 (5)	
H17	0.810392	−0.354934	0.502276	0.074*	
C16	0.7448 (2)	−0.18100 (19)	0.54090 (9)	0.0632 (5)	
H16	0.626763	−0.212940	0.527475	0.076*	
C6	−0.0776 (2)	0.59977 (17)	0.68582 (8)	0.0501 (4)	
C5	−0.1410 (2)	0.71063 (18)	0.71118 (9)	0.0558 (4)	
H5	−0.252302	0.714786	0.695759	0.067*	
C4	−0.0391 (2)	0.81545 (17)	0.75948 (9)	0.0538 (4)	
C31	0.9056 (3)	1.6916 (2)	1.09726 (13)	0.0897 (7)	
H31	0.821394	1.711933	1.066993	0.108*	
C32	1.0097 (3)	1.7978 (3)	1.14393 (15)	0.1065 (9)	
H32	0.996215	1.889094	1.144422	0.128*	
Cl	0.37075 (6)	1.23731 (8)	1.13083 (2)	0.0967 (2)	
H6	−0.146 (2)	0.5267 (19)	0.6546 (9)	0.057 (5)*	
H2	0.303 (2)	0.6937 (18)	0.7740 (9)	0.061 (5)*	
H4	−0.080 (2)	0.8914 (19)	0.7778 (9)	0.057 (5)*	

### Atomic displacement parameters (Å^2^)

**Table:**

	*U*^11^	*U*^22^	*U*^33^	*U*^12^	*U*^13^	*U*^23^
O7	0.0531 (6)	0.0670 (7)	0.0505 (6)	0.0008 (5)	0.0086 (5)	−0.0123 (5)
O6	0.0596 (7)	0.0465 (6)	0.0544 (6)	0.0017 (5)	0.0134 (5)	−0.0115 (5)
O2	0.0542 (7)	0.0636 (8)	0.0892 (9)	0.0284 (6)	−0.0096 (6)	−0.0289 (6)
O4	0.0899 (9)	0.0714 (8)	0.0624 (7)	0.0493 (7)	0.0188 (6)	0.0061 (6)
O5	0.0600 (7)	0.0755 (8)	0.0566 (7)	−0.0063 (6)	0.0056 (6)	0.0044 (6)
O3	0.1290 (13)	0.0867 (10)	0.0914 (10)	0.0694 (10)	0.0423 (9)	0.0305 (9)
O1	0.0672 (8)	0.0598 (8)	0.0861 (9)	0.0257 (6)	−0.0185 (7)	−0.0296 (7)
N1	0.0917 (13)	0.0785 (12)	0.1191 (16)	0.0446 (10)	0.0413 (11)	0.0130 (11)
C33	0.0729 (13)	0.0866 (16)	0.0667 (12)	0.0043 (11)	−0.0038 (10)	−0.0185 (11)
C34	0.0696 (12)	0.0989 (17)	0.0638 (12)	−0.0017 (11)	−0.0122 (9)	0.0277 (11)
C35	0.0744 (12)	0.0626 (11)	0.0720 (12)	0.0052 (9)	0.0026 (10)	0.0236 (9)
C30	0.0519 (9)	0.0587 (10)	0.0518 (9)	0.0056 (7)	0.0090 (7)	−0.0046 (7)
C29	0.0636 (10)	0.0634 (11)	0.0573 (10)	−0.0031 (8)	0.0135 (8)	−0.0061 (8)
C26	0.0488 (8)	0.0514 (9)	0.0461 (8)	0.0120 (7)	0.0033 (6)	−0.0037 (7)
C25	0.0622 (10)	0.0569 (10)	0.0510 (9)	−0.0031 (8)	0.0164 (8)	−0.0036 (7)
C24	0.0620 (10)	0.0524 (9)	0.0458 (8)	0.0062 (7)	0.0139 (7)	−0.0049 (7)
C23	0.0461 (8)	0.0443 (8)	0.0471 (8)	0.0146 (6)	0.0034 (6)	0.0019 (6)
C22	0.0464 (8)	0.0473 (8)	0.0490 (8)	0.0142 (7)	0.0053 (7)	0.0046 (7)
C3	0.0507 (8)	0.0411 (8)	0.0476 (8)	0.0066 (6)	0.0103 (7)	−0.0032 (6)
C2	0.0439 (8)	0.0497 (9)	0.0487 (8)	0.0123 (7)	0.0051 (7)	−0.0034 (7)
C1	0.0470 (8)	0.0447 (8)	0.0433 (8)	0.0145 (6)	0.0046 (6)	−0.0011 (6)
C7	0.0513 (9)	0.0517 (9)	0.0515 (9)	0.0187 (7)	−0.0001 (7)	−0.0068 (7)
C8	0.0485 (9)	0.0568 (10)	0.0656 (10)	0.0224 (7)	−0.0064 (8)	−0.0173 (8)
C13	0.0616 (10)	0.0555 (10)	0.0678 (11)	0.0238 (8)	0.0021 (8)	0.0005 (8)
C12	0.0581 (10)	0.0600 (10)	0.0575 (9)	0.0249 (8)	0.0076 (8)	−0.0016 (8)
C11	0.0477 (8)	0.0524 (9)	0.0474 (8)	0.0189 (7)	−0.0041 (6)	−0.0060 (7)
C14	0.0588 (10)	0.0564 (10)	0.0518 (9)	0.0239 (8)	−0.0010 (7)	−0.0045 (8)
C15	0.0700 (11)	0.0565 (10)	0.0516 (9)	0.0327 (8)	0.0109 (8)	0.0006 (7)
C20	0.0719 (12)	0.0497 (10)	0.0717 (11)	0.0170 (8)	0.0076 (9)	−0.0112 (8)
C19	0.0564 (10)	0.0588 (11)	0.0800 (12)	0.0158 (8)	0.0080 (9)	−0.0055 (9)
C18	0.0646 (10)	0.0527 (10)	0.0578 (9)	0.0240 (8)	0.0177 (8)	0.0026 (8)
C21	0.0734 (12)	0.0601 (11)	0.0766 (12)	0.0275 (9)	0.0263 (10)	0.0079 (9)
C27	0.0433 (8)	0.0724 (11)	0.0414 (8)	0.0163 (7)	0.0051 (6)	−0.0021 (7)
C28	0.0406 (8)	0.0688 (10)	0.0467 (8)	0.0082 (7)	0.0061 (6)	0.0052 (7)
C9	0.0664 (11)	0.0776 (13)	0.0630 (11)	0.0300 (9)	0.0144 (9)	−0.0054 (10)
C10	0.0645 (10)	0.0645 (11)	0.0591 (10)	0.0247 (8)	0.0076 (8)	0.0028 (8)
C17	0.0723 (11)	0.0507 (10)	0.0618 (10)	0.0198 (8)	0.0057 (8)	−0.0105 (8)
C16	0.0602 (10)	0.0657 (11)	0.0629 (10)	0.0224 (8)	−0.0004 (8)	−0.0083 (9)
C6	0.0513 (9)	0.0478 (9)	0.0492 (8)	0.0137 (7)	−0.0005 (7)	−0.0029 (7)
C5	0.0518 (9)	0.0562 (10)	0.0623 (10)	0.0225 (7)	0.0017 (7)	0.0016 (8)
C4	0.0614 (10)	0.0446 (9)	0.0594 (10)	0.0215 (7)	0.0121 (8)	−0.0013 (7)
C31	0.0782 (14)	0.0788 (14)	0.1071 (17)	0.0395 (11)	−0.0310 (12)	−0.0313 (12)
C32	0.0958 (17)	0.0809 (15)	0.133 (2)	0.0416 (13)	−0.0338 (16)	−0.0495 (15)
Cl	0.0578 (3)	0.1601 (6)	0.0491 (3)	−0.0054 (3)	0.0139 (2)	−0.0263 (3)

### Geometric parameters (Å, º)

**Table:**

O7—C26	1.3545 (19)	C1—C6	1.385 (2)
O7—C29	1.441 (2)	C1—C7	1.476 (2)
O6—C22	1.3591 (19)	C8—C13	1.370 (3)
O6—C3	1.4086 (17)	C8—C9	1.372 (3)
O2—C7	1.3563 (19)	C13—C12	1.382 (2)
O2—C8	1.396 (2)	C13—H13	0.9300
O4—C14	1.350 (2)	C12—C11	1.386 (2)
O4—C15	1.4052 (19)	C12—H12	0.9300
O5—C22	1.1953 (19)	C11—C10	1.385 (2)
O3—C14	1.190 (2)	C11—C14	1.477 (2)
O1—C7	1.1909 (19)	C15—C20	1.363 (3)
N1—C21	1.141 (2)	C15—C16	1.371 (2)
C33—C32	1.349 (3)	C20—C19	1.376 (3)
C33—C34	1.360 (3)	C20—H20	0.9300
C33—H33	0.9300	C19—C18	1.381 (2)
C34—C35	1.393 (3)	C19—H19	0.9300
C34—H34	0.9300	C18—C17	1.382 (3)
C35—C30	1.378 (3)	C18—C21	1.441 (2)
C35—H35	0.9300	C27—C28	1.370 (2)
C30—C31	1.363 (3)	C27—Cl	1.7284 (15)
C30—C29	1.495 (2)	C28—H28	0.9300
C29—H29A	0.9700	C9—C10	1.376 (3)
C29—H29B	0.9700	C9—H9	0.9300
C26—C25	1.384 (2)	C10—H10	0.9300
C26—C27	1.388 (2)	C17—C16	1.372 (2)
C25—C24	1.379 (2)	C17—H17	0.9300
C25—H25	0.9300	C16—H16	0.9300
C24—C23	1.376 (2)	C6—C5	1.378 (2)
C24—H24	0.9300	C6—H6	0.929 (18)
C23—C28	1.390 (2)	C5—C4	1.380 (2)
C23—C22	1.471 (2)	C5—H5	0.9300
C3—C2	1.369 (2)	C4—H4	0.940 (18)
C3—C4	1.373 (2)	C31—C32	1.376 (3)
C2—C1	1.392 (2)	C31—H31	0.9300
C2—H2	0.944 (18)	C32—H32	0.9300
			
C26—O7—C29	115.72 (12)	C8—C13—H13	120.5
C22—O6—C3	118.29 (12)	C12—C13—H13	120.5
C7—O2—C8	117.25 (12)	C13—C12—C11	120.16 (16)
C14—O4—C15	117.53 (13)	C13—C12—H12	119.9
C32—C33—C34	119.68 (19)	C11—C12—H12	119.9
C32—C33—H33	120.2	C10—C11—C12	119.62 (15)
C34—C33—H33	120.2	C10—C11—C14	118.11 (16)
C33—C34—C35	120.33 (19)	C12—C11—C14	122.22 (15)
C33—C34—H34	119.8	O3—C14—O4	122.49 (15)
C35—C34—H34	119.8	O3—C14—O4	122.49 (15)
C30—C35—C34	119.8 (2)	O3—C14—C11	125.25 (16)
C30—C35—H35	120.1	O4—C14—C11	112.23 (15)
C34—C35—H35	120.1	O4—C14—C11	112.23 (15)
C31—C30—C35	118.56 (17)	C20—C15—C16	122.31 (16)
C31—C30—C29	119.80 (18)	C20—C15—O4	117.50 (16)
C35—C30—C29	121.44 (18)	C16—C15—O4	120.08 (16)
O7—C29—C30	109.61 (13)	C20—C15—O4	117.50 (16)
O7—C29—H29A	109.7	C16—C15—O4	120.08 (16)
C30—C29—H29A	109.7	C15—C20—C19	118.63 (16)
O7—C29—H29B	109.7	C15—C20—H20	120.7
C30—C29—H29B	109.7	C19—C20—H20	120.7
H29A—C29—H29B	108.2	C20—C19—C18	120.22 (17)
O7—C26—C25	124.66 (14)	C20—C19—H19	119.9
O7—C26—C27	117.23 (13)	C18—C19—H19	119.9
C25—C26—C27	118.10 (14)	C19—C18—C17	120.04 (16)
C24—C25—C26	120.76 (15)	C19—C18—C21	118.90 (17)
C24—C25—H25	119.6	C17—C18—C21	121.06 (16)
C26—C25—H25	119.6	N1—C21—C18	177.7 (2)
C23—C24—C25	120.81 (14)	C28—C27—C26	121.24 (14)
C23—C24—H24	119.6	C28—C27—Cl	119.84 (12)
C25—C24—H24	119.6	C26—C27—Cl	118.91 (12)
C24—C23—C28	118.75 (14)	C27—C28—C23	120.32 (14)
C24—C23—C22	122.69 (14)	C27—C28—H28	119.8
C28—C23—C22	118.56 (14)	C23—C28—H28	119.8
O5—C22—O6	122.68 (14)	C8—C9—C10	119.15 (17)
O5—C22—O6	122.68 (14)	C8—C9—H9	120.4
O5—C22—C23	125.70 (14)	C10—C9—H9	120.4
O6—C22—C23	111.61 (13)	C9—C10—C11	120.26 (18)
O6—C22—C23	111.61 (13)	C9—C10—H10	119.9
C2—C3—C4	122.09 (14)	C11—C10—H10	119.9
C2—C3—O6	117.58 (14)	C16—C17—C18	119.79 (16)
C4—C3—O6	120.24 (14)	C16—C17—H17	120.1
C2—C3—O6	117.58 (14)	C18—C17—H17	120.1
C4—C3—O6	120.24 (14)	C15—C16—C17	119.01 (17)
C3—C2—C1	118.53 (15)	C15—C16—H16	120.5
C3—C2—H2	120.9 (11)	C17—C16—H16	120.5
C1—C2—H2	120.5 (11)	C5—C6—C1	120.23 (15)
C6—C1—C2	119.99 (14)	C5—C6—H6	120.9 (11)
C6—C1—C7	117.75 (14)	C1—C6—H6	118.8 (11)
C2—C1—C7	122.20 (14)	C6—C5—C4	119.93 (15)
O1—C7—O2	122.12 (14)	C6—C5—H5	120.0
O1—C7—O2	122.12 (14)	C4—C5—H5	120.0
O1—C7—C1	126.23 (15)	C3—C4—C5	119.21 (15)
O2—C7—C1	111.65 (13)	C3—C4—H4	119.5 (11)
O2—C7—C1	111.65 (13)	C5—C4—H4	121.3 (11)
C13—C8—C9	121.82 (16)	C30—C31—C32	121.0 (2)
C13—C8—O2	118.33 (17)	C30—C31—H31	119.5
C9—C8—O2	119.73 (16)	C32—C31—H31	119.5
C13—C8—O2	118.33 (17)	C33—C32—C31	120.6 (2)
C9—C8—O2	119.73 (16)	C33—C32—H32	119.7
C8—C13—C12	118.98 (17)	C31—C32—H32	119.7
			
C32—C33—C34—C35	0.5 (4)	C15—O4—C14—C11	170.88 (14)
C33—C34—C35—C30	−1.6 (3)	C10—C11—C14—O3	−7.1 (3)
C34—C35—C30—C31	1.5 (3)	C12—C11—C14—O3	170.25 (19)
C34—C35—C30—C29	176.40 (17)	C10—C11—C14—O4	174.84 (15)
C26—O7—C29—C30	−179.10 (15)	C12—C11—C14—O4	−7.8 (2)
C31—C30—C29—O7	−92.2 (2)	C10—C11—C14—O4	174.84 (15)
C35—C30—C29—O7	93.0 (2)	C12—C11—C14—O4	−7.8 (2)
C29—O7—C26—C25	−3.6 (3)	C14—O4—C15—C20	−98.2 (2)
C29—O7—C26—C27	175.84 (15)	C14—O4—C15—C16	85.5 (2)
O7—C26—C25—C24	178.60 (17)	C16—C15—C20—C19	−0.3 (3)
C27—C26—C25—C24	−0.9 (3)	O4—C15—C20—C19	−176.45 (16)
C26—C25—C24—C23	0.1 (3)	O4—C15—C20—C19	−176.45 (16)
C25—C24—C23—C28	0.2 (3)	C15—C20—C19—C18	0.2 (3)
C25—C24—C23—C22	−179.69 (16)	C20—C19—C18—C17	0.4 (3)
C3—O6—C22—O5	−0.7 (2)	C20—C19—C18—C21	−178.57 (18)
C3—O6—C22—C23	−179.61 (13)	O7—C26—C27—C28	−178.18 (15)
C24—C23—C22—O5	173.94 (17)	C25—C26—C27—C28	1.3 (3)
C28—C23—C22—O5	−6.0 (3)	O7—C26—C27—Cl	0.5 (2)
C24—C23—C22—O6	−7.2 (2)	C25—C26—C27—Cl	179.98 (14)
C28—C23—C22—O6	172.84 (14)	C26—C27—C28—C23	−1.0 (3)
C24—C23—C22—O6	−7.2 (2)	Cl—C27—C28—C23	−179.65 (13)
C28—C23—C22—O6	172.84 (14)	C24—C23—C28—C27	0.2 (2)
C22—O6—C3—C2	−110.45 (16)	C22—C23—C28—C27	−179.86 (15)
C22—O6—C3—C4	72.9 (2)	C13—C8—C9—C10	0.1 (3)
C4—C3—C2—C1	−0.3 (2)	O2—C8—C9—C10	−175.81 (15)
O6—C3—C2—C1	−176.95 (13)	O2—C8—C9—C10	−175.81 (15)
O6—C3—C2—C1	−176.95 (13)	C8—C9—C10—C11	0.3 (3)
C3—C2—C1—C6	−0.1 (2)	C12—C11—C10—C9	−0.3 (3)
C3—C2—C1—C7	−177.12 (15)	C14—C11—C10—C9	177.15 (16)
C8—O2—C7—O1	−3.1 (3)	C19—C18—C17—C16	−0.8 (3)
C8—O2—C7—C1	176.36 (15)	C21—C18—C17—C16	178.11 (17)
C6—C1—C7—O1	−2.8 (3)	C20—C15—C16—C17	−0.2 (3)
C2—C1—C7—O1	174.35 (17)	O4—C15—C16—C17	175.92 (16)
C6—C1—C7—O2	177.76 (14)	O4—C15—C16—C17	175.92 (16)
C2—C1—C7—O2	−5.1 (2)	C18—C17—C16—C15	0.7 (3)
C6—C1—C7—O2	177.76 (14)	C2—C1—C6—C5	0.3 (2)
C2—C1—C7—O2	−5.1 (2)	C7—C1—C6—C5	177.48 (15)
C7—O2—C8—C13	99.95 (19)	C1—C6—C5—C4	−0.1 (3)
C7—O2—C8—C9	−84.0 (2)	C2—C3—C4—C5	0.5 (3)
C9—C8—C13—C12	−0.6 (3)	O6—C3—C4—C5	177.03 (14)
O2—C8—C13—C12	175.35 (14)	O6—C3—C4—C5	177.03 (14)
O2—C8—C13—C12	175.35 (14)	C6—C5—C4—C3	−0.3 (3)
C8—C13—C12—C11	0.7 (2)	C35—C30—C31—C32	−0.3 (4)
C13—C12—C11—C10	−0.2 (2)	C29—C30—C31—C32	−175.3 (2)
C13—C12—C11—C14	−177.55 (15)	C34—C33—C32—C31	0.7 (4)
C15—O4—C14—O3	−7.2 (3)	C30—C31—C32—C33	−0.9 (4)

### Hydrogen-bond geometry (Å, º)

*Cg*4 and *cg*5 are the centroids of the C23–C28 and C30–C35 rings, respectively.

**Table:**

*D*—H···*A*	*D*—H	H···*A*	*D*···*A*	*D*—H···*A*
C2—H2···O2	0.944 (18)	2.411 (17)	2.7213 (19)	98.9 (12)
C12—H12···O4	0.93	2.42	2.733 (2)	100
C24—H24···O6	0.93	2.40	2.721 (2)	100
C17—H17···N1^i^	0.93	2.62	3.504 (3)	158
C25—H25···*Cg*5^ii^	0.93	2.86	3.744 (2)	158
C31—H31···*Cg*4^iii^	0.93	2.82	3.702 (3)	158

Symmetry codes: (i) −*x*+2, −*y*−1, −*z*+1; (ii) −*x*, −*y*−1, −*z*; (iii) −*x*+1, −*y*−1, −*z*.
